# Reviewing the Emergence of* Lactococcus garvieae*: A Case of Catheter Associated Urinary Tract Infection Caused by* Lactococcus garvieae* and* Escherichia coli* Coinfection

**DOI:** 10.1155/2017/5921865

**Published:** 2017-07-24

**Authors:** Tatvam T. Choksi, Farhan Dadani

**Affiliations:** ^1^Department of Hospital Medicine, The University of Chicago Medical Center, 5841 S. Maryland Avenue, Chicago, IL 60637, USA; ^2^Mercy Hospital and Medical Center, 2525 S. Michigan Avenue, Chicago, IL 60616, USA

## Abstract

*Lactococcus garvieae* is considered a low virulence organism which is rarely associated with human infections. Most of the reported cases have been associated with bacteremia with or without endocarditis. We report a rare case of catheter associated urinary tract infection (CAUTI) caused by* Lactococcus garvieae* and* Escherichia coli* coinfection without any bacteremia in a patient with indwelling urinary catheter placed for benign prostatic hyperplasia (BPH). The patient also had a history of gastroesophageal reflux disease (GERD) with long standing famotidine treatment. In our case,* Lactococcus garvieae* was initially misidentified as* Enterococcus* species but was later detected by automated microbial identification system VITEK-2 (BioMerieux, Marcy-l'Étoile, France) and was confirmed with API 32 Strep system (BioMerieux, Marcy-l'Étoile, France). The patient responded well to a two-week course of cephalosporin. Interestingly, apart from the acid suppressive treatment, no other risk factors were identified.

## 1. Introduction

*Lactococcus *species have traditionally been recognized as low virulence organisms causing opportunistic infections in humans [1, 2]. But, in recent years, steadily increasing number of human cases associated with* Lactococcus garvieae* are being reported. Among these cases, the majority have been associated with bacteremia with infective endocarditis [3–14], although liver abscess [15], spontaneous septicemia [6], bone involvement [1, 16, 17], diverticulitis [6], and secondary peritonitis [6] have also been described. From our review of literature, we found one case of urinary tract sepsis caused by* Lactococcus garvieae* and this patient had* L. garvieae* bacteremia as a complication of transurethral prostatic resection [18]. We report a rare case of catheter associated urinary tract infection caused by the organism without any bacteremia in a patient with indwelling urinary catheter.

## 2. Case

A 78-year-old African American male with history of hypertension, well controlled diabetes mellitus type II, gastroesophageal reflux disease (GERD) on famotidine, and morbid obesity initially presented to our institution with lower urinary tract symptoms 4 weeks ago. He was diagnosed with benign prostatic hyperplasia (BPH), was started on tamsulosin plus finasteride, and was discharged with outpatient follow-up with urology. Two weeks later, on follow-up with his urologist, he was noted to have significant urinary retention and so an indwelling Foley catheter was placed. Ten days later, he started noticing hematuria after having a mechanical fall. So, he presented again to our institution's emergency department. His urine analysis (UA) showed gross hematuria with more than 100 red blood cells (RBC) per high power field (hpf) and moderate bacteriuria, but no leukocyte esterase or nitrites were noted. His vital signs were stable with no fever or tachycardia. Urine culture was sent and he was discharged following continuous bladder irrigation. Urine culture later revealed contamination with 3 or more different species.

Forty-eight hours later, the patient returned to the emergency department with persistent hematuria and suprapubic tenderness. He was noted to have a temperature of 38.4 degrees Celsius, a heart rate of 102 beats per minute, and a white blood count (WBC) of 12.8 × 10^9^/L with neutrophilic predominance of 80.1%. A UA performed demonstrated a red turbid specimen with more than 100 RBC/hpf and more than 100 WBC/hpf and moderate bacteriuria with 3+ leukocyte esterase. Physical exam was remarkable for suprapubic tenderness. Laboratory data showed lactic acid of 2.2 mg/dL and serum Cr of 2.3 mg/dL (increased from a baseline of 1.8 mg/dL).

The Foley catheter was replaced. Urine culture and two sets of blood cultures were sent. The patient was resuscitated with intravenous fluids and started on intravenous vancomycin 1.5 gram 12 hourly and cefepime 1 gram 8 hourly based on creatinine clearance. Chest radiograph and abdominal examination were unremarkable and he demonstrated no other sites of infection. Both sets of blood cultures remained negative. On day 2 of admission, the urine culture on colistin nalidixic acid (CNA) gram-positive medium at 37 degrees Celsius was reported to be growing >100,000 colony forming units per milliliter (cfu/mL) of* Enterococcus* species, while that on MacConkey agar grew >100,000 cfu/ml of* Escherichia coli*. On day 4 of hospitalization, the microbiology lab reported that the* Enterococcus* species was reidentified as* Lactococcus garvieae*. Initially utilizing the PYR (Pyrrolidonyl Arylamidase) test (Remel Inc., Lenexa, KS), the* Enterococcus* genus was identified and the automated VITEK-2 system (BioMerieux, Marcy-l'Étoile, France; VITEK 2 AST GP67 cards) was deployed for further speciation which reidentified the organism as* Lactococcus garvieae *(>99% probability). Furthermore, the sample was also run on an API 32 Strep system (BioMerieux, Marcy-l'Étoile, France) and confirmed the isolate as* Lactococcus garvieae*. There are no specific breakpoints available for antimicrobial susceptibility/resistance for* L. garvieae*. Antibiotic selection was done based on the zones of inhibition by Kirby-Bauer method. The diameter of the inhibition zone for penicillin was 22 mm and that for third-generation cephalosporin was 20 mm, while the diameter for inhibition zones for clindamycin, bacitracin, and gentamycin was 0 mm suggestive of high resistance.

The patient showed rapid improvement on the initial broad spectrum antibiotic regimen for the first 3 days which was changed on day 4 to intravenous ceftriaxone 1 gram every 24 hours. He remained hospitalized for a week for getting placed to a subacute rehab facility. He was finally discharged on cefpodoxime 100 mg every 12 hours for another 7 days. His repeat urine culture demonstrated clearance of infection. He remained in good health at the subacute rehab facility on follow-up.

Upon questioning, the patient and his family denied any consumption of raw fish, seafood, or unpasteurized milk. But they reported having canned food on regular basis.

## 3. Discussion

In 1985, based on genetic evidence of DNA-DNA relatedness and DNA-RNA relatedness, the genus* Lactococcus* was separated from the genus* Streptococcus* [19].* Lactococci* are gram-positive, catalase-negative, facultatively anaerobic cocci that occur singly, in pairs, or in chains and produce lactic acid from fermentation of carbohydrates. They comprise 8 different species and subspecies. Most species are not associated with human disease [18]; but in recent years,* L. garvieae* has been increasingly recognized as an emerging zoonotic pathogen [12, 20].

The review of literature, till date, suggests some similarities among patients infected by* L. garvieae*. The majority of cases had bacteremia [3–18, 21]. Among these, many presented as infective endocarditis of either native or prosthetic valves [3–14, 21], while the others had bacteremia associated with liver abscess [15], diverticulitis [6], spondylitis [16, 17], or multiorgan dysfunction [6]. A case of bacteremia from urinary tract infection after TURP has been reported [18]. Cases of secondary peritonitis from ruptured jejunum [6] and primary infective spondylodiscitis [1] have also been reported. Also, the organism has been noted to be causing coinfection with* K. pneumoniae* and* Enterococcus* [1, 6]. But no case report of* L. garvieae* and* E. coli* coinfection has been reported so far. Here we describe the unique case of CAUTI caused by* L. garvieae* and* E. coli* coinfection. No bacteremia was noted. While* E. coli* is a usual pathogen associated with urinary tract infections, it is unclear how the patient acquired* L. garvieae*.

From the review of reported cases, an association between raw fish consumption and human infection is noted [1, 6, 12, 17]. Contaminated milk and unpasteurized dairy products can also serve as the potential source [12, 21, 22]. Moreover, they have also been isolated in manufactured food due to their use in food products [12]. Other risk factors include anatomical or physiological abnormalities of gastrointestinal tract secondary to previous surgeries, on-going intestinal pathology, or consumption of acid suppressive medications [1]. It was noted that those with multiple comorbidities and significant systemic illness suffered a more complicated course [1]. Our patient was on long standing famotidine treatment for his acid reflux disease but did not suffer from any gastrointestinal pathology and denied consumption of any raw fish or unpasteurized dairy products. But he admitted having canned food and processed meat on regular basis.

As seen in our case, identification of* Lactococcus* species can be challenging. This is not only because both* Lactococcus* and* Enterococcus* species share some phenotypical features, but also due to the fact that* Lactococcus garvieae* tend to be PYR positive [14, 19, 23]. Hence, it can be easily misidentified initially as* Enterococcus* species. Automated microbial identification systems, such as the VITEK-2, have been helpful for identification of the organism but the results should be confirmed with a different method [14]. We used API 32 Strep system for confirmation as mentioned in the case. Clindamycin susceptibility testing further facilitates the differentiation between* Lactococcus lactis* and* Lactococcus garvieae* [12, 18]. Other methods of identification include genetic testing with 16S ribosomal RNA and sodAint genes [8, 18]. Besides the clindamycin test, a PCR assay based on primers deduced from the regions carrying the 16S ribosomal RNA genes of* L. garvieae* is available which can differentiate* L. garvieae* from* L. lactis* [20]. Traditionally, combination of biochemical testing, VITEK-2, and API 32 Strep system have been used for identification of* Lactococcus* species; but discordance among these tests can happen and so genetic testing with 16S r-RNA and/or PCR testing may be necessary. But they are expensive and, like at our medical center, these tests are often not available in many medical centers worldwide and may contribute to the underdiagnosis of* L. garvieae* infections [12].

Penicillins and cephalosporins both are usually effective in treatment of* Lactococcus garvieae* infections [24]. Most of the patients with endocarditis have been treated with 6 weeks of penicillin or ampicillin with or without 2 weeks of gentamycin [1, 18]. Our patient responded well to a course of 14 days of third-generation cephalosporin treatment.

## 4. Conclusion

Human infections caused by* L. garvieae* are extremely rare but lately it has started to become associated with a variety of infections. Though now exact pathogenesis remains unclear, over a period with more reported cases, we should have more insight into the area. Diagnosis of* Lactococcus garvieae* infection may pose a challenge due to the organism's shared phenotypical features with* Enterococcus* species, and, therefore, when genetic testing or PCR is not available, combination of the traditional testing should be used to correctly identify the organism.

## References

[1] Chan J. F. W., Woo P. C. Y., Teng J. L. L. (2011). Primary infective spondylodiscitis caused by *Lactococcus garvieae* and a review of human *L. garvieae* infections. *Infection*.

[2] Collins M. D., Farrow J. A., Phillips B. A., Kandler O. (1983). *Streptococcus garvieae* sp. nov. and *Streptococcus plantarum* sp. nov. *Microbiology*.

[3] Rawling R. A., Granato P. A. (2014). *Lactococcus garvieae* native valve endocarditis. *Clinical Microbiology Newsletter*.

[4] Fefer J. J., Ratzan K. R., Sharp S. E., Saiz E. (1998). *Lactococcus garvieae* endocarditis: report of a case and review of the literature. *Diagnostic Microbiology and Infectious Disease*.

[5] Vinh D. C., Nichol K. A., Rand F., Embil J. M. (2006). Native-valve bacterial endocarditis caused by *Lactococcus garvieae*. *Diagnostic Microbiology and Infectious Disease*.

[6] Wang C.-Y. C., Shie H.-S., Chen S.-C. (2007). *Lactococcus garvieae* infections in humans: possible association with aquaculture outbreaks. *International Journal of Clinical Practice*.

[7] Li W.-K., Chen Y.-S., Wann S.-R., Liu Y.-C., Tsai H.-C. (2008). *Lactococcus garvieae* endocarditis with initial presentation of acute cerebral infarction in a healthy immunocompetent man. *Internal Medicine*.

[8] Fihman V., Raskine L., Barrou Z. (2006). *Lactococcus garvieae* endocarditis: identification by 16S rRNA and sodA sequence analysis. *Journal of Infection*.

[9] Yiu K.-H., Siu C.-W., To K. K.-W. (2007). A rare cause of infective endocarditis: *Lactococcus garvieae*. *International Journal of Cardiology*.

[10] Wilbring M., Alexiou K., Reichenspurner H., Matschke K., Tugtekin S. M. (2011). *Lactococcus garvieae* causing zoonotic prosthetic valve endocarditis. *Clinical Research in Cardiology*.

[11] Zuily S., Mami Z., Meune C. (2011). *Lactococcus garvieae* endocarditis. *Archives of Cardiovascular Diseases*.

[12] Hirakawa T. F., da Costa F. A. A., Vilela M. C., Rigon M., Abensur H., de Araújo M. R. E. (2011). Lactococcus garvieae endocarditis: first case report in Latin America. *Arquivos Brasileiros de Cardiologia*.

[13] Fleming H., Fowler S. V., Nguyen L., Hofinger D. M. (2012). Lactococcus garvieae multi-valve infective endocarditis in a traveler returning from South Korea. *Travel Medicine and Infectious Disease*.

[14] Navas M. E., Hall G., El Bejjani D. (2013). A case of endocarditis caused by *Lactococcus garvieae* and suggested methods for identification. *Journal of Clinical Microbiology*.

[15] Mofredj A., Baraka D., Cadranel J. F., LeMaitre P., Kloeti G., Dumont J. L. (2000). *Lactococcus garvieae* septicemia with liver abscess in an immunosuppressed patient. *The American Journal of Medicine*.

[16] James P. R., Hardman S. M. C., Patterson D. L. H. (2000). Osteomyelitis and possible endocarditis secondary to *Lactococcus garvieae*: a first case report. *Postgraduate Medical Journal*.

[17] Aubin G. G., Bémer P., Guillouzouic A. (2011). First report of a hip prosthetic and joint infection caused by *Lactococcus garvieae* in a woman fishmonger. *Journal of Clinical Microbiology*.

[18] Dylewski J. (2014). Urinary Tract Sepsis Caused by *Lactococcus garvieae*. *Clinical Microbiology Newsletter*.

[19] Facklam R., Elliott J. A. (1995). Identification, classification, and clinical relevance of catalase-negative, gram-positive cocci, excluding the *streptococci* and *enterococci*. *Clinical Microbiology Reviews*.

[20] Zlotkin A., Eldar A., Ghittino C., Bercovier H. (1998). Identification of *Lactococcus garvieae* by PCR. *Journal of Clinical Microbiology*.

[21] Russo G., Iannetta M., D'Abramo A. (2012). Lactococcus garvieae endocarditis in a patient with colonic diverticulosis: first case report in Italy and review of the literature. *New Microbiologica*.

[22] Fortina M. G., Ricci G., Foschino R. (2007). Phenotypic typing, technological properties and safety aspects of *Lactococcus garvieae* strains from dairy environments. *Journal of Applied Microbiology*.

[23] Schleifer K. H., Kraus J., Dvorak C. (1985). Transfer of *Streptococcus Lactis* and related *Streptococci* to the genus *Lactococcus* gen. *Systematic and Applied Microbiology*.

[24] Elliott J. A., Facklam R. R. (1996). Antimicrobial susceptibilities of *Lactococcus lactis* and *Lactococcus garvieae* and a proposed method to discriminate between them. *Journal of Clinical Microbiology*.

